# Expression-based network biology identifies immune-related functional modules involved in plant defense

**DOI:** 10.1186/1471-2164-15-421

**Published:** 2014-06-03

**Authors:** Joel P Tully, Aubrey E Hill, Hadia MR Ahmed, Ryan Whitley, Anthony Skjellum, M Shahid Mukhtar

**Affiliations:** Department of Computer & Information Sciences, University of Alabama at Birmingham, Birmingham, AL 35294-1170 USA; Samuel Ginn College of Engineering, 3101 Shelby Center, Auburn University, Auburn, AL 36849-5347 USA; Department of Biology, University of Alabama at Birmingham, Birmingham, AL 35294-1170 USA; Nutrition Obesity Research Center, University of Alabama at Birmingham, Birmingham, AL 35294 USA

**Keywords:** Pathogen and pathogen-mimic stimuli, Linear and non-linear models, Transcriptional regulatory network, Network topology, GO terms, OCCEAN software, Immune-related functional modules

## Abstract

**Background:**

Plants respond to diverse environmental cues including microbial perturbations by coordinated regulation of thousands of genes. These intricate transcriptional regulatory interactions depend on the recognition of specific promoter sequences by regulatory transcription factors. The combinatorial and cooperative action of multiple transcription factors defines a regulatory network that enables plant cells to respond to distinct biological signals. The identification of immune-related modules in large-scale transcriptional regulatory networks can reveal the mechanisms by which exposure to a pathogen elicits a precise phenotypic immune response.

**Results:**

We have generated a large-scale immune co-expression network using a comprehensive set of *Arabidopsis thaliana* (hereafter Arabidopsis) transcriptomic data, which consists of a wide spectrum of immune responses to pathogens or pathogen-mimicking stimuli treatments. We employed both linear and non-linear models to generate Arabidopsis immune co-expression regulatory (AICR) network. We computed network topological properties and ascertained that this newly constructed immune network is densely connected, possesses hubs, exhibits high modularity, and displays hallmarks of a “real” biological network. We partitioned the network and identified 156 novel modules related to immune functions. Gene Ontology (GO) enrichment analyses provided insight into the key biological processes involved in determining finely tuned immune responses. We also developed novel software called OCCEAN (*O*ne *C*lick *Cis*-regulatory *E*lements *AN*alysis) to discover statistically enriched promoter elements in the upstream regulatory regions of Arabidopsis at a whole genome level. We demonstrated that OCCEAN exhibits higher precision than the existing promoter element discovery tools. In light of known and newly discovered *cis*-regulatory elements, we evaluated biological significance of two key immune-related functional modules and proposed mechanism(s) to explain how large sets of diverse GO genes coherently function to mount effective immune responses.

**Conclusions:**

We used a network-based, top-down approach to discover immune-related modules from transcriptomic data in Arabidopsis. Detailed analyses of these functional modules reveal new insight into the topological properties of immune co-expression networks and a comprehensive understanding of multifaceted plant defense responses. We present evidence that our newly developed software, OCCEAN, could become a popular tool for the Arabidopsis research community as well as potentially expand to analyze other eukaryotic genomes.

**Electronic supplementary material:**

The online version of this article (doi:10.1186/1471-2164-15-421) contains supplementary material, which is available to authorized users.

## Background

Agriculture, in struggling to meet global food demands of a rapidly growing population, has been plagued with plant diseases that thwart crop production and account for a global annual average yield loss of 16 percent [1–3]. In light of the importance of agriculture to humans, it is essential to elucidate and understand the mechanisms of plant immunity at the molecular level. As with any host-pathogen conflict, plants and their disease agents are in an evolutionary arms race. When the host mounts a defense reaction, the pathogen develops new strategies to evade the defensive mechanisms, which causes the continuation of this cycle, *ad infinitum*[4, 5]. Our present understanding of plant immune systems reveals two primary means by which plants recognize their invaders. The recognition of Microbial-Associated Molecular Patterns (MAMPs) is the first line of defense and its associated MAMPs-Triggered Immunity (MTI) is highly efficient at repelling most pathogens. If pathogens are able to breach this first line of defense through the production of effector proteins, plants deploy Effector-Triggered Immunity (ETI) [4, 6]. Both modes of defense cause massive transcriptional reprogramming which involves complex signal transduction networks and cross-talk that is mediated by plant hormones, including: Salicylic Acid (SA), Jasmonic Acid (JA) and Ethylene (ET) [7, 8]. The Arabidopsis genome encodes approximately 1,922 transcription factors (TFs) that are implicated in diverse biological processes including the regulation of immune signaling pathways [9–11]. However, the global organization of TF-DNA interactions remains elusive. Moreover, what remains mostly unknown are the precise mechanisms by which the plant cell integrates innumerable synergistic and antagonistic immune transcriptomic signals to orchestrate fine-tuned and pathogen-specific defense responses.

In recent years, systems biology approaches, specifically network analyses that integrate experimentally derived information with computational modeling, have emerged as powerful tools for studying complex traits in diverse species [5, 12–15]. Network biology provides comprehensive analyses of the system’s components (nodes) and the relationships among them (edges). In addition, the analyses of the topological properties of the network can provide further understanding of the hierarchical organization of a complex biological system and contribute to the overall interpretation of biological complexity. In any eukaryotic cell, thousands of genes and their products orchestrate their transcriptional and translational activities to ensure the proper execution of cellular functions [13, 15, 16]. It has become evident that biological functions can be accomplished by functional modules that are embedded within the interaction networks including transcriptional gene regulatory networks [17, 18]. Comprehensive high-throughput analyses of gene expression can allow for the identification of gene clusters that are highly correlated in expression levels across multiple samples in any given cellular state [13, 16]. Generally, it is thought that genes in the same co-expression sub-network are often enriched with similar functional annotations. Additionally, the metric to measure the co-expression falls into one of two major categories: correlation coefficients or mutual information measures [19]. Finally, finding common *cis*-regulatory elements (transcription factor binding sites) can aid in the identification of co-regulated gene clusters and characterization of transcriptional regulatory networks [13, 14, 17].

In the current study we employed a systems biology approach and report the construction of a large-scale Arabidopsis immune co-expression regulatory (AICR) network. We tested five diverse algorithms, *i.e.* PCC (Pearson Correlation Coefficient), ARACNE multiplicative (Algorithm for the Reconstruction of Accurate Cellular Networks), ARACNE additive, CLR (Context Likelihood of Relatedness), and MRNET (Minimum Redundancy NETwork) with different thresholds, which yielded 15 pairs of experimental networks along with their respective random networks [19–25]. We employed network biology analyses and determined that ARACNE multiplicative network (5,147 nodes and 38,610 edges), with threshold of 0.8 exhibits properties of a “true” network as it possesses the scale-freeness attribute (degree distribution follows a power law). Next, we partitioned the AICR network and predicted 156 functional modules containing at least six members with the largest module encompassing 178 nodes. Subsequently, we analyzed functional annotations of genes within each module and calculated enrichment of specific Gene Ontology terms to evaluate the biological significance of functional modules. To establish a causal relationship between co-expression and co-regulation, we sought to identify common *cis*-regulatory elements. First we developed a new, comprehensive software interface for *cis*-regulatory elements discovery in Arabidopsis (named OCCEAN - One Click *Cis*-regulatory Elements ANalysis). We demonstrated that OCCEAN boasts higher capacity (can process the entire genome scale; over 30 million characters in a single run) and features higher precision than MEME (Multiple EM for Motif Elicitation). We identified several statistically enriched novel *cis*-regulatory elements in our dataset. Finally, we evaluated and discussed key immune-related functional modules.

## Results and discussion

### Construction of the Arabidopsis immune co-expression regulatory (AICR) network

**Figure 1:**
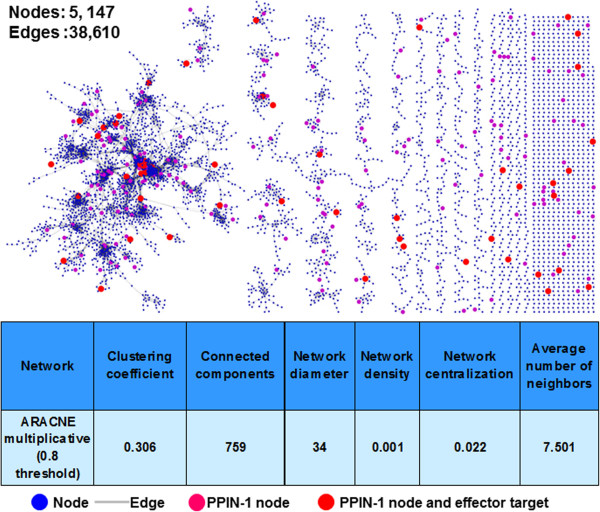
**Construction of Arabidopsis immune co-expression regulatory (AICR) network.** Network is displayed in Prefuse Force Directed Layout algorithm in Cytoscape. A node (blue circle) represents genes and a grey edge linking two nodes indicates the co-expression relationship between these two nodes based on Pearson Correlation Coefficient (PCC). Red nodes represent the proteins present in Plant-pathogen Interaction network (PPIN-1) [5]. The common feature of experimental the AICR network is listed on the bottom.

To generate a large-scale immune co-expression network, we selected Arabidopsis transcriptomic data that encompassed the broadest possible spectrum of immune responses to pathogens or pathogen-mimicking stimuli treatments [26–34]. Previously, we have employed the same set of experiments to define transcriptional responses of genes that encode the proteins identified in plant-pathogen immune network, version 1′ (PPIN-1) [5] (Additional file 1: Table S1 and Additional file 2: Supporting methods). We compiled the lists of probes showing significant up- or down-regulation and discovered 8,377 differentially expressed genes, which subsequently were used to build a comprehensive immune co-expression network (Additional file 3: Table S2). In a co-expression network, the nodes represent the genes and the edges (lines connecting nodes) represent the similarity/relatedness between the genes, which is generally measured by the Pearson correlation coefficient (PCC) [18]. PCC and other measures of association determine the degree of linear dependencies between the variables [19]. While these association models have obvious statistical advantages, they lack the ability to capture non-linear relationships [35]. In contrast, mutual information (MI) is a non-linear statistic that provides an attractive alternative to measure biologically significant non-linear relationships [36]. We used both linear and non-linear models to generate Arabidopsis immune co-expression regulatory (AICR) network. To measure linear associations between two genes, we calculated the PCC values for all of the genes in a matrix of 8,377 × 8,377 (over 70 million combinations). Then, we employed both a similarity threshold algorithm (a predefined single cut-off value) as well as a newly developed and parameter-free algorithm termed mutual *k*-nearest neighbor (mKNN) to process the calculated PCC values [18]. mKNN was recently shown to possess several advantages over the commonly used similarity threshold algorithm [18]. Using the linear model, we generated seven pairs of experimental networks along with their respective random networks, *i.e.,* PCC (0.9), K3, K10, K20, K50, K100 and K250 (Table 1). To infer non-linear association between immune-related genes, we employed four recently used MI methods: ARACNE multiplicative (Algorithm for the Reconstruction of Accurate Cellular Networks), ARACNE additive, CLR (Context Likelihood of Relatedness), and MRNET (Minimum Redundancy NETwork) with 0.8 and 0.9 thresholds. Specifically, we employed the ‘parmigene’ package (PARallel Mutual Information estimation for GEne NEtwork reconstruction, an R package) to construct eight Additional experimental networks [21–25]. It has been recently shown that the MI estimator implemented in parmigene provides more precision and unbiased results compared with previous MI estimators (Figure 1). Our approach to initially build multiple experimental and random networks using both linear and non-linear models is aimed to determine an optimal experimental network that displays topological properties of a “real” biological network [15].

**Table 1:** **Comparison of multiple algorithms used to generate Arabidopsis Immune Co-expression (AICR) Network**

Algorithm	Threshold	Nodes	Edges
PCC K = 3	Top 3	8250	1371822
PCC K = 10	Top 10	8336	1374677
PCC K = 20	Top 20	8358	1379968
PCC K = 50	Top 50	8369	1396874
PCC K = 100	Top 100	8373	1453118
PCC K = 250	Top 250	8377	1758775
PCC	90.00%	8377	2755191
ARACNE additive	90.00%	1999	10253
ARACNE multiplicative	90.00%	1999	10253
MRNET	90.00%	1999	3493
CLR	90.00%	12	11
ARACNE additive	80.00%	5147	32972
ARACNE multiplicative	80.00%	5147	38610
MRNET	80.00%	5058	6049
CLR	80.00%	57	60

*PCC*, pearson correlation coefficients; *ARACNE*, algorithm for the reconstruction of accurate cellular networks; *CLR*, context likelihood of relatedness; *MRNET*, minimum redundancy NETwork.

### Global topological properties of the AICR network

**Figure 2:**
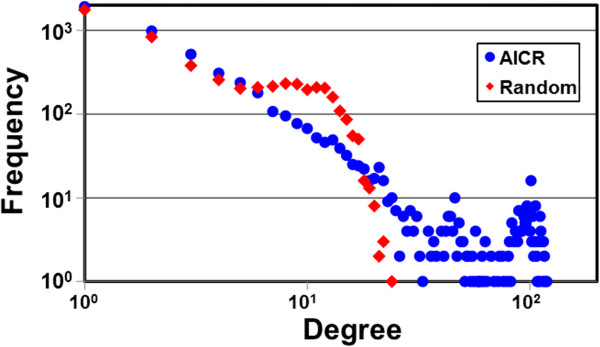
**Degree distribution of the AICR.** Frequency of the degree of the AICR (blue circles) and random (red diamonds) networks are indicated in log scale. Presence of highly connected nodes (hubs) can be observed in the AICR network.

Network topology refers to the arrangement or pattern of interactions within a network. The majority of naturally occurring networks, including biological networks, maintain certain topological characteristics in terms of their structure and organization that are significantly different from random networks [15, 37, 38]. Therefore, in an initial experiment, we computed degree of distribution for all 15 pairs of experimental networks along with their respective random networks. We found that two networks, ARACNE multiplicative (5,147 nodes and 38,610 edges) and ARACNE additive (5,147 nodes and 32,972 edges), with threshold of 0.8 exhibit the scale-freeness attribute (degree distribution follows a power law), when compared with all random networks and other experimental networks (Figure 2). Since the precision of parmigene-based ARACNE multiplicative is better than ARACNE additive, we selected ARACNE multiplicative with threshold 0.8 as the “true” experimental AICR network [25].

**Figure 3:**
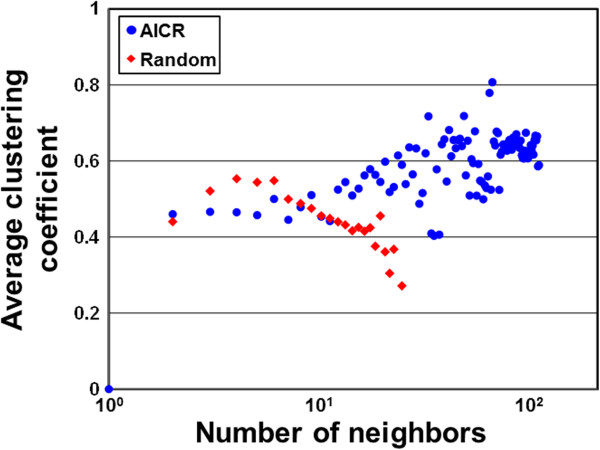
**Average clustering coefficient of the AICR.** A plot between number of neighbors (X-axis) and average clustering coefficient (Y-axis) is drawn representing the AICR (blue circles) and random (red diamonds) networks.

**Figure 4:**
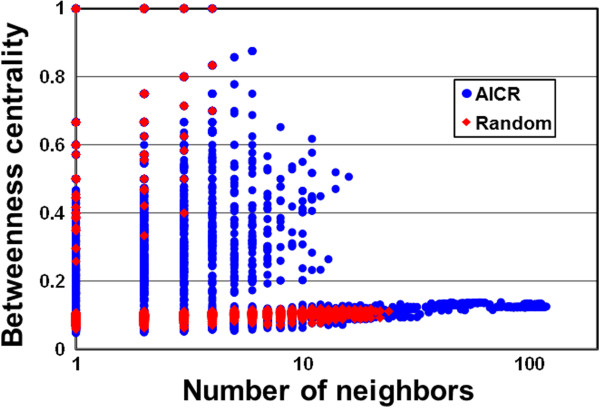
**Betweenness centrality of the AICR.** Frequency of Betweenness centrality of the AICR (blue circles) and random (red diamonds) networks are indicated.

“Real-world networks” tend to form high-density clusters and exhibit a clustering coefficient that is significantly higher than expected by random chance [14, 18]. Therefore, we computed clustering coefficients of the AICR network and its corresponding random network (Figure 3). A clustering coefficient describes the degree of congregation among the nodes of a graph. The distribution of the average clustering coefficient in the AICR ranges between 0.4-0.8 and the frequency of the number of neighbors with higher clustering coefficient in the AICR is significantly greater than in a random network (Figure 3). To understand how the differences in the number of connections impact the topology of our co-expression network, we measured the shortest path (shortest distance between all pairs of nodes) and the closeness centrality (the inverse sum of shortest distances to all other nodes from a focal node) (Additional file 4: Figure S1 and Additional file 5: Figure S2) [39]. The path lengths for the majority of the nodes in the AICR are significantly shorter than those of a random network (Additional file 4: Figure S1). In addition, the number of neighbors with significantly higher closeness centrality (threshold 0.2) is higher in the AICR network compared with random network (Additional file 5: Figure S2). In biological networks, highly connected nodes (hubs) and nodes central to the network (betweenness) are thought to play significant regulatory roles on their adjacent nodes. Thus, we computed several topological properties including shared neighbor distribution (number of interaction partners shared between two nodes) (Additional file 6: Figure S3), neighborhood connectivity distribution (average connectivity of all neighbors) (Additional file 7: Figure S4), topological coefficients (the tendency of the nodes in the network to have shared neighbors) (Additional file 8: Figure S5) and betweenness centrality (nodes’ centrality in a network) (Figure 4) [37, 38]. These data suggest that the AICR network is not randomly organized and shares properties of several previously described biological networks [5, 12–15]. Another important characteristic of scale-free networks is the presence of a main component. Connected component analyses discovered that the AICR network comprises 63 disconnected components, each containing at least six members. The main component in the AICR network has 2379 nodes and 33214 (86%) edges. The second largest component contains only 79 nodes and 310 edges. This qualitative global topology of the AICR network is similar to previously known biological networks in Arabidopsis and other eukaryotes [14, 40]. In addition, network biology analyses revealed that degree distribution of main component of the AICR follows a power law compared to the main component of a random network with similar size (Additional file 9: Figure S6 and Additional file 10: Figure S7).

### Biological implications of topological properties of the AICR network

Topological properties of the network can also be utilized to prioritize genes for further functional analysis. Highly connected and highly centered nodes are likely to play crucial roles in maintaining network integrity and controlling information flow throughout the plant immune system. Specifically, we identified 190 highly connected nodes (Hub^50^ encompassing at least 50 connections in the AICR). To characterize the significance of these highly connected proteins, we combined Hub^50^ of the AICR with biophysical interactions from plant-pathogen immune network, version 1′ (PPIN-1; Figure 1) [5]. The PPIN-1 was constructed using effectors (virulence factors) from two pathogens, Arabidopsis immune proteins, and ~8,000 other Arabidopsis proteins covering almost one-third of the genome [5]. Pathogens utilize effector molecules to rewire the host network in a manner conducive to pathogen proliferation and dispersal [41]. PPIN-1 discovered 165 effector interacting proteins (effector targets) and it was also demonstrated that these effector targets are highly interconnected host proteins [5]. Despite the unavailability of effector targets from the entire Arabidopsis genome in PPIN-1, we found that the AICR network contains 51 effector targets and three proteins (At3g63210, At4g11890 and At3g07780) are Hub^50^ in the AICR network (Figure 1). Given that transcriptional regulation does not necessarily coincide with biophysical interactions, this overlap is potentially significant and biologically meaningful. This also suggests that pathogen effectors target key regulatory proteins in both transcriptional as well as protein-protein interactions networks.

In addition, we selected ninety nodes (“top 90”) with the highest betweeness centrality (Figure 4). Among them, we found a number of previously characterized immune regulators or defense-related proteins, encompassing various levels of signal transduction flow: resistance proteins (RPP4, uncharacterized TIR-NBS-LRR) [42, 43], kinases mediating early signaling events (MAPKKK13, WAK) [44], transcriptional executors (ANAC072, WRKY54, MYB66, WER1) [45–47], secretory proteins assisting with folding and modification of newly synthesized proteins (TRAP, CNX3, Sec14p, cyclophilin, UDP-Glycosyltransferase, DnaJ family, KMS1, GlcNAc1pUT2) [48, 49], enzymes involved in production of phytohormones and antimicrobial compounds (AOC2, ILL3, PAL2) and pathogenesis-related proteins (PR1) [50, 51], hormonal response modulators (PYL6, IAA28) [52, 53] and finally, components of ubiquitin-mediated protein degradation (UBQ4, an F-box protein that interacts with SKP1) [54, 55]. Identification of these key immune regulators serves as a proof of concept for our analytical approach and justifies further analyses on suite of “top 90” proteins selected among thousands of other proteins in the AICR network.

### Network clustering and module annotations

Another essential feature of a majority of biological networks is their ability to naturally organize into modules [14, 17, 18]. Discovery of such functional modules within biological networks is imperative for understanding principles of cellular organization and functions. To identify functional modules (subnetworks) that are comprised of highly interconnected sets of genes within the AICR network, we employed a fast agglomerative algorithm (FAG-EC) [56]. This new, low time complexity algorithm operates based on a local variable, edge clustering coefficients, making it ideal to partition a relatively large network [37, 56]. Overall, we identified 156 immune-related functional modules containing at least six members with the largest module encompassing 178 nodes (Additional file 11: Table S3). Furthermore, we subjected the AICR network to a module size distribution analysis. We computed the frequency and module size and demonstrated that the distribution of the module size follows a power law distribution with an exponential truncation (Additional file 12: Figure S8), which is common for several previously described “real-world” networks [14, 18]. The module size distribution property further indicates that the AICR network possesses a modular structure.

We also investigated the enrichment of Gene Ontology (GO) terms [57, 58] in the ten largest immune-related modules (“top 10”) with sizes ranging from 38 to 178 distinct nodes using the Database for Annotation, Visualization and Integrated Discovery (DAVID) v6.7 [59] (Additional file 13: Table S4). The GO enrichment terms are remarkably informative in examining significant biological functions among co-expressed genes in large-scale networks [58, 59]. Several gene ontology (GO) categories were enriched (p-values >0.01) among the genes in the AICR network (Table 2). These major GO categories include: immune-related, plastid, reproductive processes, nucleotide binding/kinases, intrinsic to membrane, metal-binding, mitochondrion and non-membrane-bounded organelle. We utilized these GO enrichments in combination with significantly enriched *cis*-regulatory elements to define the potential functions of the immune-related modules (described below).

**Table 2:** **Significantly enriched GO terms in the ten largest immune-related modules with sizes ranging from 38 to 178 genes**

Module	Go term	Count	%	P value	Fold enrichment
1	GO:0000166 ~ nucleotide binding	46	25.84	6.946003862092387E-5	1.732
1	GO:0006796 ~ phosphate metabolic process	31	17.42	5.723269851412882E-7	2.705
1	GO:0004672 ~ protein kinase activity	29	16.29	2.6741028021510666E-8	3.278
1	GO:0008219 ~ cell death	14	7.87	4.2878037087740953E-7	6.123
2	GO:0009536 ~ plastid	31	30.69	9.044388056227762E-4	1.748
2	GO:0004672 ~ protein kinase activity	12	11.88	0.007513	2.463
2	GO:0019748 ~ secondary metabolic process	8	7.92	0.003083	4.073
3	GO:0009536 ~ plastid	36	49.32	3.047542263866877E-10	2.718
3	GO:0055114 ~ oxidation reduction	11	15.07	0.002191	3.019
4	GO:0005886 ~ plasma membrane	16	22.22	0.001229	2.356
4	GO:0006793 ~ phosphorus metabolic process	14	19.44	9.785189944953485E-5	3.418
5	GO:0009628 ~ response to abiotic stimulus	9	15.52	0.009708	2.845
5	GO:0005618 ~ cell wall	5	8.62	0.056662	3.316
6	GO:0009507 ~ chloroplast	22	44.00	1.0752825917609916E-5	2.499
6	GO:0051186 ~ cofactor metabolic process	7	14.00	2.8760079199003342E-5	10.975
7	GO:0000166 ~ nucleotide binding	12	25.53	0.044134	1.777
8	GO:0009725 ~ response to hormone stimulus	8	17.39	0.001339	4.400
8	GO:0006793 ~ phosphorus metabolic process	6	13.04	0.093212	2.354
9	GO:0031224 ~ intrinsic to membrane	13	33.33	0.002493	2.407
9	GO:0006793 ~ phosphorus metabolic process	8	20.51	0.008887	3.139
10	GO:0032555 ~ purine ribonucleotide binding	10	26.32	0.083444	1.765
10	GO:0005886 ~ plasma membrane	8	21.05	0.052468	2.151

### Development of OCCEAN software and identification of *cis*-regulatory elements

**Figure 5:**
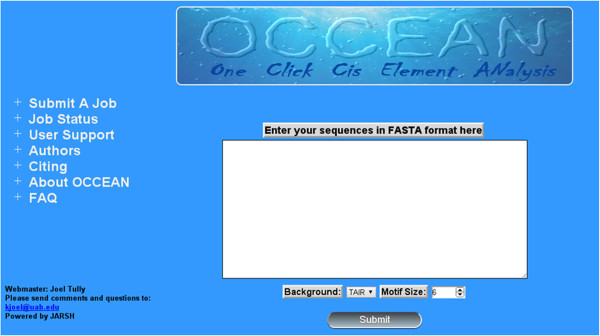
**Screenshot of OCCEAN (** ***O*** **ne** ***C*** **lick** ***Cis*** **-regulatory** ***E*** **lements** ***AN*** **alysis).** Results can be obtained as an excel sheet and Additional information can be accessed in pop-up windows.

**Figure 6:**
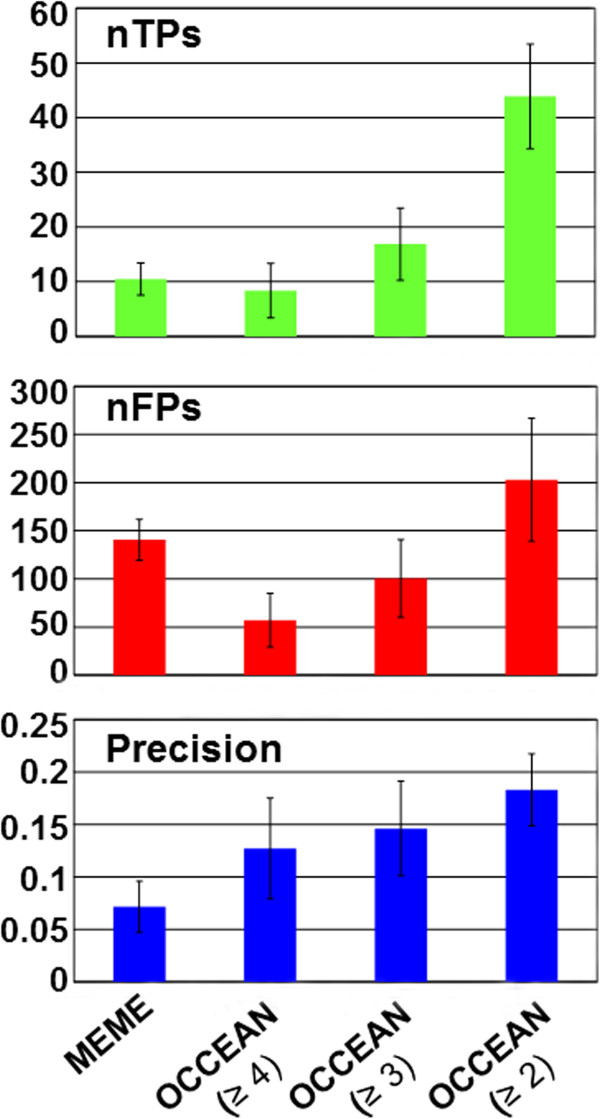
**Performance and accuracy of OCCEAN.** True positives (nTPs), false positives (nFPs) and precision for MEME as well as OCCEAN with multiple enrichment ratios are shown.

We sought to investigate whether all or a subset of the co-expressed genes within a given immune-related module are also co-regulated (*i.e.,* are direct targets of a common transcription factor). Given that transcriptional regulatory networks are highly complex and that functional modules may display crosstalk among themselves, we can also expect that the same transcription factor can regulate co-expressed genes in multiple immune-related modules. Whereas experimentally verified plant *cis*-regulatory elements can be retrieved from PLACE (A Database of Plant *Cis*-acting Regulatory DNA Elements) [60], Athena (Arabidopsis thaliana expression network analysis) [61] and AGRIS (The Arabidopsis Gene Regulatory Information Server) [11, 62], only ~120 *cis*-regulatory elements are currently known in Arabidopsis, and a limited set of ~30 immune-related *cis*-elements have been described. In addition, the prediction of *cis*-elements from classical approaches is typically driven by a single experiment or dataset. Moreover, the currently available online motif discovery software has limited capacity to process a small number of gene entries. For example, frequently used software MEME (Multiple EM for Motif Elicitation) can only process up to 60,000 characters in a single run (*e.g.,* 60 promoters, each of 1,000 bp in length) [63, 64]. Another common software used to predict *cis*-regulatory elements, AthaMap (Arabidopsis thaliana Map) can only manage up to 200 gene entries [65]. These limitations can substantially decrease the chance of identifying novel *cis*-regulatory elements. Thus, we first aimed to develop OCCEAN (*O*ne-*C*lick *C*is-regulatory *E*lement *AN*alysis) software that can process the promoter gene sequences at the entire genome scale (over 30 million characters) in a single run. OCCEAN identifies the statistically enriched/depleted *cis*-regulatory elements (Figure 5) and integrates data from (*i*) our newly developed BLASTN-based program to identify short sequences and (*ii*) an improved version of the bootstrapping tool POBO (a promoter bootstrapping program) [64]. This user-friendly software requires the sequences in FASTA format as input, processes genome-scale level sequences, identifies common sequences in a given set of promoters, performs bootstrapping, and provides statistically enriched *cis*-regulatory elements in the gene’s promoter as the output. OCCEAN is freely available online at http://occean.cis.uab.edu:8080/occean/. We employed OCCEAN to individually process the sequences of immune-related genes’ promoters for our “top 10” immune-related modules. The promoter sequences of the genome (33,323 genes) were used as background to compute the fold enrichments of putative *cis*-regulatory elements. We also analyzed the occurrences of all the putative *cis*-regulatory elements (cluster mean), performed 1,000 × bootstrapping to determine the background mean and finally calculated fold enrichment ratios for all six-mer sequences. Three different fold enrichment ratios (≥4, ≥ 3 and ≥ 2) prioritized newly identified *cis*-regulatory elements in “top 10” immune-related modules (Additional file 14: Table S5). To analyze the performance and robustness of our newly developed software, we compared the efficacy of OCCEAN with MEME. We generated a list of experimentally known *cis*-regulatory elements and computed the precision of both OCCEAN and MEME using *cis*-regulatory elements identified in our top 10 immune-related modules. MEME was unable to compute the analyses on top 4 immune-related modules as it can’t process over 60,000 characters simultaneously. We computed true positives (nTPs), false positives (nFPs) and precision for MEME as well as OCCEAN enrichment ratios ≥ 4, ≥ 3 and ≥ 2 (Figure 6). We selected an enrichment ratio ≥ 3 as the optimal value for OCCEAN as it yields a higher number of nTPs, significantly fewer nFPs positives and greater precisions compared with enrichment ratios ≥ 4 and ≥ 2. In summary, we demonstrated that OCCEAN boasts higher capacity (can process the entire genome scale; over 30 million characters in a single run) and features higher precision than MEME (Figure 6). Identification of *cis*-regulatory elements among co-expressed genes could provide additional information for prediction of biological function.

### Inferring biological relevance of immune-related modules

**Figure 7:**
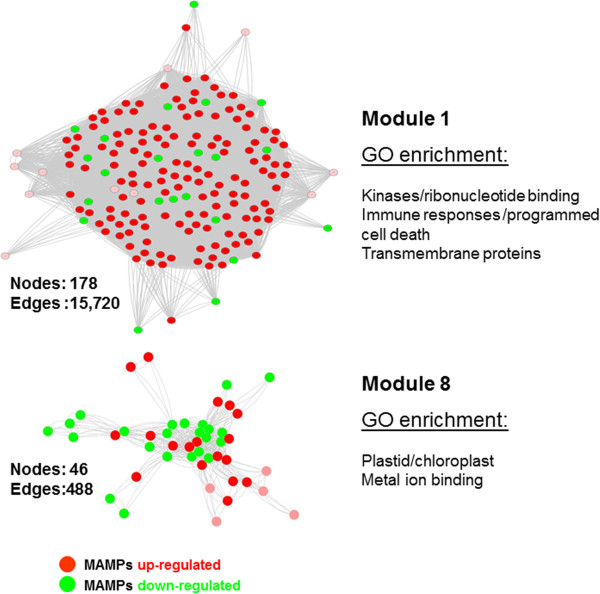
**Inferring molecular functions of the immune-related modules.** Module 1 (top) and Module 8 (bottom) is presented. Module 2 is displayed in dark and light green colors. Red and green nodes represent up-regulation and down-regulation of genes within these modules by OGs (oligogalacturonide) and flg22 (flagellin 22). The most over-represented biological process GO term was indicated with each module.

To fend off potential pathogens, plants employ two major types of immune responses: MAMPs-Triggered Immunity (MTI) and Effector Triggered Immunity (ETI) [4]. ETI is essentially a high amplitude re-booting of the immediate but weaker MTI. ETI results in robust disease resistance responses, including localized host cell death (hypersensitive response; HR) and systemic signaling [4]. In addition, upon pathogen recognition at the MTI and/or ETI levels, the plant cell undergoes extensive transcriptional changes involving complex reiterative signaling networks and cross-talk controlled by phytohormones [66]. This leads to the metabolic reprogramming and production of an array of antimicrobial compounds [27]. Here we highlight two major immune-related functional modules, the largest identified Module 1 (Figure 7, Additional file 11: Table S3 and Additional file 13: Table S4) and a more compact Module 8 (Figure 7, Additional file 15; Supporting results, Additional file 11: Table S3 and Additional file 13: Table S4) as two most representative gene clusters reflecting the broad array of diverse pathogens and immune stimuli used in the microarray experiments employed in our analyses. Module 1 is the largest module identified by our clustering analyses and comprises 178 nodes, 15,720 edges, clustering coefficient of 0.692 and network density of 0.499. The genes of Module 1 are enriched in three major GO categories: (1) kinases/ribonucleotide binding, (2) immune responses/programmed cell death and (3) transmembrane proteins.

Diverse kinases account for the major functional category of Module 1, encompassing 46 out of 178 proteins representing receptor-like kinases (RLKs), mitogen-activated protein kinases (MAPKs), calcium-dependent protein kinases (CDPKs), wall-associated kinases (WAKs), etc. Typically, RLKs perceive MAMPs such as flg22, elf18, chitin and OGs and trigger MTI [27]. Subsequently, a MAPK cascade is activated and downstream signaling ensues including phytohormonal crosstalk [8, 27, 44, 67]. These events result in the activation of a wide-spectrum of immune responses, such as induced biosynthesis of phytoalexins and other antimicrobial secondary metabolites such as glucosinolates, calcium influx, as well as production and accumulation of reactive oxygen species (ROS) [27, 68].

At the second, more powerful layer of the immune response, plants usually deploy NLR receptors (nucleotide-binding domain and leucine-rich repeat receptors; a major class of R proteins). The NLR receptors directly recognize specific effectors or indirectly detect effector activities and trigger ETI [1, 2, 6, 7]. We discovered 8 *NLR* genes in the kinase sub-cluster of Module 1. These include proteins conferring resistance to diverse pathogens such as *Pseudomonas syringae, Albugo candida, Hyaloperonospora arabidopsidis* and various *Fusarium* races.

The second most abundant GO category in Module 1 was immune-related genes (count: 32). This functional sub-cluster contains various defense-related transcription factors such as WRKY15, WRKY33, MYB113 and ANAC061 [9, 47], four members of the RING-H2 finger protein family, a heat shock transcription factor HSF A-4a [53], an ethylene-responsive transcription factor ERF105 and, intriguingly, three scarecrow-like GRAS family transcription factor genes, known to be primarily implicated in plant root development [69–71]. Collectively, transcription factor genes account for over 40% of this sub-cluster’s gene count. In addition, we identified a small group of genes strongly associated with response to fungal cell wall elicitor chitin, such as two auxin-regulated SAUR genes, one lectin-like, Enhanced Disease Susceptibility 1 (EDS1) and senescence-associated protein SAG102 [72–76].

Another enriched GO category in Module 1 was membrane-associated proteins including both plasma membrane- and organellar membrane-resident peptides; not surprisingly, the largest class of these membrane proteins is transporters. In plants, uptake and translocation of nutrients play essential roles in physiological processes including plant growth, nutrition, signal transduction, and development [77–79]. Transport processes are also critical for the reallocation of resources and it was previously shown that defense signals cause realignment of transport activities to redirect resources toward immune responses and protect tissues of high value [80]. Of 31 genes assigned to this sub-cluster, seven were transport proteins (22.6% of this sub-cluster’s gene count). The prevailing class was sugar (galactose and sucrose) transporters, in agreement with the fact that this class of proteins constitutes a key component for carbon partitioning at the whole plant level and is involved in both symbiotic and parasitic plant-fungi interactions [81]. Additionally, we identified *CNI1* (*Carbon-Nitrogen Insensitive 1*) that is a key regulator of the Carbon/Nitrogen response for growth phase transition in Arabidopsis seedlings [82], as well as ATP Binding Cassette (ABC) and inorganic phosphate transporters that were previously postulated to be required for organ growth, nutrition, development, and stress responses [83]. Last but not least, we also identified two other well-known immune regulators: *Syntaxin 121/PEN1* and *MLO2* genes, required for resistance against barley powdery mildew, *Blumeria graminis* sp. *hordei* and a fungal pathogen *Colletotrichum higginsianum*[84, 85].

Another essential aspect of large-scale transcriptomic analyses is to link co-expression with co-regulation, *i.e.,* to determine presence of common *cis*-regulatory elements among genes of the same module. Several known *cis*-regulatory elements were discovered in the promoters of Module 1 genes (Additional file 14: Table S5). Two of the enriched elements, *TCATGG* and *CATGGA*, overlap with the octadecameric CArG box sequence 5′-*CTTACCTTTCATGGATTA*-3′, identified in the *APETALA3* promoter [86]. *APETALA3*, a class B homeotic organ identity gene, was originally discovered as the central regulator of petal development in Arabidopsis flowers, but later also shown to be involved in control of the floral meristem proliferation, regulation of flowering time, and other plant reproductive processes [87–89]. Inhibition of reproductive processes is an expected outcome of an immune stimulation, given the recent report describing a transition from growth to defense following immune stimuli treatments in Arabidopsis [53]. Involvement of a developmental regulator in defense responses is not unprecedented and was previously shown for a MYB-related gene *ASYMMETRIC LEAVES 1* (*AS1*) [90].

Jasmonic Acid (JA) and Ethylene (ET) are two plant hormones antagonistic to SA [91] and down-regulation of major JA/ET signaling proteins will promote SA signaling by suppressing the JA/ET pathway [67]. Consistent with this observation, we identified an enriched element, *ATCTTG* that resembles the binding site for Ethylene-Insensitive 3 (EIN3) and three EIN3-LIKE (EIL) proteins [92] as well as two hexamers *GTCGTC* and *CGTCGT*, which overlap with the core binding site of *JASE2* motif in two JA biosynthetic genes *OPR1/OPR2*[93]. Recently, EIN3 and EIL1 were shown to negatively regulate MAMP-triggered immunity via a direct binding and down-regulation of the SA biosynthetic gene *SID2*[94]. Given that OPRs are implicated in JA biosynthesis, we expect a fine-tuned interplay between SA and JA/ET signaling pathways [95]. In addition, several novel *cis*-regulatory elements were discovered in our study (Additional file 14: Table S5), and the identity of the cognate transcription factors and their functional relevance will be subject to future studies. Very recently, it was demonstrated that numerous transcription factors can recognize secondary elements, which in some cases are completely sequence-unrelated to the primary element [96]. In conjunction with this study, our analysis of Module 1 shows that ARR14 (a MYB family transcription factor) not only recognizes the known primary *cis*-regulatory element *AGATA/TCG* but also can specifically bind to a previously unknown *cis*-regulatory element *AGATCT*. Thus, it’s likely that transcription factor(s) have extended list of binding specificities beyond the currently known *cis*-regulatory elements. We propose that the above described transcription factors and additional unknown regulatory proteins coordinate the gene regulation in this module.

Our data provide further insights into the transcriptional regulatory mechanisms repressing additional putative negative regulators of plant defense upon treatments with pathogen-mimicking stimuli.

## Conclusions

In this study, we used a systems-level network biology approach to construct genome-wide Arabidopsis immune co-expression network and demonstrated that this network shares properties of a ‘real-world network’. Topological properties-based partitioning allowed us to unravel 156 distinct immune-related functional modules. We demonstrated that nodes in the same module are not only highly correlated at the expression level but also densely connected to each other. Detailed analyses of two key immune-related modules provided a systems-level understanding of how plant cells coordinate distinct immune signals to orchestrate fine-tuned and pathogen-specific defense responses. Our novel approach to discover *cis*-regulatory elements using OCCEAN is an effective method of solving the issue of finding novel motifs within a sequence set. OCCEAN has advantages over other programs of the same purpose, such as APPLES (Analysis of Plant Promoter-Linked Elements) and MEME [63, 97]. APPLES requires finding organisms of a specified relational distance for comparison, which can burden the user, and MEME has the statistical risk of discarding actual existing motifs. These problems are avoided in our solution whilst continuing to maintain a fair amount of focus on client-side simplicity. In addition, OCCEAN has the capacity to be expanded for analyses of other eukaryotes genomes, such as fly and human. Our systems-level approach to examine *cis*-regulatory elements (the putative transcription factor binding sites) in the promoters of the co-expressed genes made it possible to link co-expression to co-regulation of genes in the same module.

## Methods

### Data download, selection criteria for differentially expressed (DE) genes dataset and promoter sequence acquisition

We utilized available transcriptomic data of transcriptional responses extracted from 271 microarray experiments representing nine major immune-related studies (Additional file 1: Table S1 and Additional file 2: Supporting methods) [26–34]. Priority was given to well-referenced studies, employing the Affymetrix ATH1 GeneChip array, encompassing the broadest possible spectrum of plant defense responses upon pathogen infections or pathogen-mimicking stimuli treatment (Additional file 2: Supporting methods). Lists of probes showing significant up- or down-regulation in each experimental condition were compiled, using criteria for significance employed in the respective original study (Additional file 3: Table S2). This led us to the identification of a list 8,377 genes differentially expressed between all treatments [5]. For each of these genes, we acquired 1000 bp upstream of the transcriptional start site from TAIR Version 10 at http://www.arabidopsis.org[98]. These upstream regions were searched for putative transcription factor binding sites.

### Network construction, topological properties, network clustering and Gene Ontology (GO)

The microarray data presented in Additional file 3: Table S2 was used to construct a gene co-expression network using both linear and non-linear models. In the linear model, Pearson Correlation Coefficients (PCC) was measured based on the mutual k-nearest neighbor method of Ruan et al. [18, 97] with some modifications. In contrast to Ruan et al. [18, 97], the k-list for each gene included k-1 other genes, and at least one (and possibly more) gene sharing the smallest of the k PCCs. For example, for a k of 10, the k-list might include 9 genes in order of decreasing PCC and one or more genes having the next lower PCC value. This was done in consideration of the fact that all genes of equal PCC are equally valid as connected nodes. Random networks were constructed by randomly assigning gene expression values (0 = no change, +1 = up-regulated, -1 = down-regulated) to the genes from the immune transcriptional profiling experiments. The network based on PCC-threshold was inferred by first calculating the PCC for every gene-gene pair in the dataset yielding 8,377 × 8,377 PCC values. These values were tested against 0.9 threshold and only the PCC values that were greater than or equal to 0.9 were selected to indicate that there is an edge between this gene pairs. Other values were discarded. R was used to calculate the non-linear mutual information (MI) [99]. First the MI was calculated using parmigene package were the MI for every gene pair was calculated resulting in 8377 × 8377 MI values [25]. Then these values where used by the ARACNE multiplicative (Algorithm for the Reconstruction of Accurate Cellular Networks), ARACNE additive, CLR (Context Likelihood of Relatedness), and MRNET (Minimum Redundancy NETwork) algorithms, using the same package, to infer a weighted adjacency matrix [21–25]. 80% and 90% of the maximum MI values in each weight adjacency matrix were chosen as the threshold to determine the existence of an edge between each pair of genes. If the value is greater than or equal to the threshold, this indicates an edge between a given pair of genes.

A modified and parallelized Cytoscape version 2.8.4 [37, 38] and the Clusterviz version 1.2 (FAG-EC algorithm) [37, 38, 56, 100] and ClusterMaker version 1.10 [100, 101] plugins were used to visualize this network, calculate its parameters and network topological properties. Network partitioning was performed using FAG-EC algorithm [56, 100] and the Markov Clustering Algorithm [102]. For each gene in the network sub-clusters, a Gene Ontology [57, 58] molecular function and biological process was assigned from the Database for Annotation, Visualization and Integrated Discovery (DAVID) v6.7 [59].

BLAST-based generation of common *cis*-regulatory elements Sequences obtained from TAIR [98] (1000 bp 5′ of the transcription start site) were incorporated into a local BLAST [103, 104] database using the formatdb utility that is included in NCBI’s package download of BLAST. Each sequence in the local BLAST database was used in turn as a query sequence to find subsequences shared in the promoter regions of a number of genes. The resulting common subsequences were imported into a relational database. Similar sequences with at least 75% identity and an E-value ≤ 1× 10^-4^ were scanned using a sliding window search in order to extract all contained 6-mers. All 6-mers having no more than four consecutive single nucleotide repeats were deemed putative transcription factor binding sites and retained for further computational analysis. This process has been incorporated into our web-accessible tool, OCCEAN.

### Statistical enrichment of *cis*-regulatory elements using modified version of POBO

Putative transcription factor binding sites results from BLAST were analyzed for statistically significant over representation/under representation using the POBO (a promoter bootstrapping program) tool [64]. POBO is an exceptional tool for statistical sequence significance analysis, but is inhibited by the constraint that it can perform analysis only on one *cis*-regulatory element at a time. As our sequence sets were high in number, manual entry would be infeasible. Therefore, a wrapper program was written to take each individual sequence, create a cluster file consisting of the set of promoter identifiers/sequences from the original experimentally found gene promoter dataset that contain the sequence, and run an instance of POBO with the sequence and cluster file as input. The program then extracted the desired information from the resulting HTML files from all of the individual runs and put them in a single spreadsheet for analysis. Running POBO locally required downloading the source code from http://ekhidna.biocenter.helsinki.fi/poxo/pobo/ and setting up a local MySQL database with all known Arabidopsis gene promoters corresponding to ~33,000 genes as the background data. 1 kb promoter sequence for all ~33,000 genes were obtained from the TAIR10 genome release, and used as background for the POBO analysis. The POBO results were converted to spreadsheet format for further analysis. True positives (nTPs), false positives (nFPs) and precision for MEME as well as OCCEAN were calculated as described in [25].

### Development of One-Click Cis-Element ANalysis (OCCEAN) software for genome-wide identification of *cis*-regulatory elements

The bulk of OCCEAN on the server-side was developed in the Java language (version 7) using Apache Tomcat (version 7, as well) on a Linux server. A Java Servlet interface was used to communicate with OCCEAN’s web interface. OCCEAN automates and integrates the information obtained from BLAST and POBO analysis. This user friendly software requires promoter sequences in FASTA format. Background sequences of the species under study are imbedded in the software. OCCEAN is time-efficient and will return a link to the file containing the results the BLAST and POBO. OCCEAN’s web interface was developed using HTML, CSS, Javascript, and AJAX for in-page update notifications.

## Electronic supplementary material

Additional file 1: Table S1: Differentially expressed (DE) genes that were determined from defense-related experiments. (XLS 26 KB)

Additional file 2: **Supporting methods Transcriptomic data of transcriptional responses extracted from 271 microarray experiments representing nine major immune-related previous studies.** (DOCX 38 KB)

Additional file 3: Table S2: AGIs of the up-regulated and down-regulated genes derived from Table S1 studies. (XLSX 253 KB)

Additional file 4: Figure S1: Distribution of shortest paths in the AICR (blue circles) and random (red diamonds) networks. (TIFF 707 KB)

Additional file 5: Figure S2: Closeness centrality property of the AICR network. Distribution of closeness centrality in the AICR (blue circles) and random (red diamonds) networks. (TIFF 1 MB)

Additional file 6: Figure S3: Evaluation of frequency of number of shared neighbors in the AICR (blue circles) and random (red diamonds) networks. (TIFF 884 KB)

Additional file 7: Figure S4: Determination of neighborhood connectivity frequency in the AICR (blue circles) and random (red diamonds) networks. (TIFF 774 KB)

Additional file 8: Figure S5: Distribution of topological coefficients in the AICR (blue circles) and random (red diamonds) networks. (TIFF 2 MB)

Additional file 9: Figure S6: Degree distributions of main components in the AICR (blue circles) and random (red diamonds) networks. (TIFF 56 KB)

Additional file 10: Figure S7: Betweenness centrality of main components in the AICR (blue circles) and random (red diamonds) networks. (TIFF 57 KB)

Additional file 11: Table S3: Identification of 156 immune-related modules. Size of each module is indicated. (XLS 146 KB)

Additional file 12: Figure S8: Distribution of module size in the AICR network. Frequency of module size in the AICR (blue circles) network is shown in log scale. The AICR network exhibits a power law distribution, a network property shared by ‘real-world networks’. (TIFF 462 KB)

Additional file 13: Table S4: GO enrichment in ten largest immune-related modules. (XLS 104 KB)

Additional file 14: Table S5: Identification of *cis*-regulatory elements in ten largest immune-related modules using OCCEAN. (XLS 128 KB)

Additional file 15: **Supporting results Module 8 in Arabidopsis immune co-expression regulatory (AICR) network.** (DOCX 34 KB)

## Abbreviations

mKNN: mutual k-nearest neighbor
GO: Gene ontology
OCCEAN: One click cis-regulatory elements ANalysis
MAMPs: Microbial-associated molecular patterns
MTI: MAMPs-triggered immunity
ETI: Effector-triggered immunity
SA: Salicylic acid
JA: Jasmonic acid
ET: Ethylene
TFs: Transcription factors
PCC: Pearson correlation coefficient
FAG-EC: Fast agglomerate algorithm- edge clustering
MEME: Multiple EM for motif elicitation
AthaMap: Arabidopsis thaliana map
PLACE: A database of plant cis-acting regulatory DNA elements
AGRIS: The arabidopsis gene regulatory information server
POBO: A promoter bootstrapping program
APPLES: Analysis of plant promoter-linked elements, nTPs, True positives
nFPs: False positives
ARACNE: Algorithm for the reconstruction of accurate cellular networks)
CLR: Context likelihood of relatedness
MRNET: Minimum redundancy NETwork
AICR: Arabidopsis immune co-expression regulatory.
