# Supplementary material for: Expression-based network biology identifies immune-related functional modules involved in plant defense
Source: BMC Genomics. 2014 Jun 3;15:421. doi: 10.1186/1471-2164-15-421 (PMC4070563; doi:10.1186/1471-2164-15-421)
Supplement: Supplementary file 2 — Additional file 2: Supporting methods Transcriptomic data of transcriptional responses extracted from 271 microarray experiments representing nine major immune-related previous studies. (DOCX 38 KB) [file 12864_2013_6122_MOESM2_ESM.docx]

**Additional file 2: Supporting methods** Transcriptomic data of transcriptional responses extracted from 271 microarray experiments representing nine major immune-related previous studies.

Our dataset encompasses a total of 271 Affymetrix microarray chips and 26 different pathogenic strains/isolates/treatments, which have been elaborated upon in the Supplemental File. These nine studies chosen for our analyses constitute the most comprehensive body of work describing diverse forms of biotrophic and hemibiotrophic infections and well as pathogen-mimicking stimuli and phytohormones involved in the immune cross-talk. Specifically, we included data on three groups of treatments:

**(1) live pathogens:** *Hyaloperonospora arabidopsidis* (biotrophic oomycete; three different isolates Emoy2, Hiks1 and Emco5), *Golovinomyces cichoracearum* (biotrophic fungus), multiple strains of *Pseudomonas syringae* (hemibiotrophic bacterium): **(a)** wild-type virulent strain DC3000 to identify the transcriptional changes associated with basal defense to live bacteria, which carry multiple MAMPs and effectors, **(b)** avirulent strain carrying an effector avrRpm1 to report induced gene-for-gene based bacterial restriction, **(c)** two different *DC3000::hrpA* and *hrpS* mutants that fail to deliver TTEs to report basal defense; **(d)** *Pst DC3118 COR^−^* mutant to identify pathogenic toxin coronatin-responsive genes, **(e)** *E. coli TUV-86-2 fliC* (a non-host pathogen of Arabidopsis) and *fliC mutants of DC3000* (to define the contribution of MAMPS to the regulation of the basal defense transcriptome during infection).

**(2)** **pathogen-mimicking stimuli:** **(a)** flg22, derived from bacterial flagellin – a potent inducer of basal defenses; **(b)** elf18 and elf26 – two variants of elicitors derived from the bacterial elongation factor EF-Tu, **(c)** chitin oligomers - components of fungal cell walls that reliably mimic early stages of responses to fungal infections, and **(d)** Oligogalacturonides (OGs; derived from plant cell wall and released by degrading enzymes during pathogenesis).

**(3) phytohormones treatments**: eight basic phytohormones (auxin, cytokinin, gibberellin, brassinosteroid, salicylic acid, abscisic acid, jasmonate and ethylene). These eight compounds are involved in a considerable amount of cross-talk that has been extensively reviewed over the last years. In summary, auxin, jasmonic acid, ethylene and abscissic acid are the best known antagonizers of salicylic acid and defense against biotrophic and hemibiotrophic pathogens.

**1- Study by Truman et al. (2006) [**[**1**](#_ENREF_1)**]**

Number of Microarrays: 27

Objectives:

-to identify the contribution to host transcriptional re-programming made by bacterial MAMPs and TTE proteins

Challenges:

-DC3000::hrpA a mutant compromised in T3SS (hrp) inoculation that fails to deliver TTEs to report basal defense,

- DC3000 to report pathogenicity and

- DC3000 delivery of the AvrRpm1 effector (which is recognized by RPM1) to report induced gene-for-gene based bacterial restriction

-mock inoculation (MgCl_2_) as a negative control to report background inoculation effects

Sampling times:

-2h, 4h and 12h; carefully chosen to reflect, as far as possible, infective stages undergoing rapid molecular responses, enabled the identification of differentially expressed genes associated with the activation of basal defense and establishment of pathogenesis.

**2- Study by Zipfel et al. (2006) [**[**2**](#_ENREF_2)**]**

Number of Microarrays: 14

Objectives:

To identify the first line of plant defense - sets of genes transcriptionally responsive to MAMPs elf18 and elf26

Challenges:

- two derivatives of bacterial elongation factor EF-Tu: elf18 and elf26

Sampling times:

0, 30 min, 60 min

**3- Study by Denoux et al. (2008) [**[**3**](#_ENREF_3)**]**

Number of Microarrays: 12

Objectives:

To identify the first line of plant defense - sets of genes transcriptionally responsive to MAMPs Oligogalacturonides (Ogs; derived from plant cell wall and released by degrading enzymes during pathogenesis) and Flg22, a fragment of the bacterial flagellin.

Challenges:

Flg22 and OGs

Sampling times:

1h, 3h

**4- Study by Eulgem et al. (2004) [**[**4**](#_ENREF_4)**]**

Objectives:

To present a comparative analysis of global gene expression patterns in response to pathogenic oomycete *Hyaloperonospora arabidopsidis*, triggered by three different *R*-dependent defense pathways: (1) the canonical *RPP4* that is dependent on PAD4, SA accumulation, and SGT1b; (2) the *RPP7* pathway that is dependent on SGT1b but independent of PAD4 or SA accumulation; and (3) the unique *RPP8* pathway that is independent of PAD4, SA accumulation, or SGT1b.

Challenges:

Three independent isolates of Hpa: Emoy2, Hiks1 and Emco5.

Sampling times:

0h, 12h, 48h

Number of Microarrays: 30, each chip reflects the average of three independent biological experiments covering approximately 150 plants and thousands of interaction sites

**5- Study by Ramonell et al. (2005) [**[**5**](#_ENREF_5)**]**

Number of Microarrays: 9

Objectives:

To perform a comprehensive analysis of transcriptional changes triggered by chitin. Chito-oligosaccharides can be generated from the cell walls of pathogenic fungi by the action of endochitinases and were shown to elicit strong defense responses in many plant species.

Challenges:

chito-octamers, hydrolyzed chitin, water (negative control)

Sampling times:

0 min, 30 min

**6- Study by Wang et al. (2006) [**[**6**](#_ENREF_6)**]**

Number of Microarrays: 27

Objectives:

A tightly controlled transcriptional profiling strategy to analyze salicylic acid-mediated and NPR1-dependent/independent plant immune responses. The unique design of this study allows studying the transcriptional events one at a time.

Challenges:

BTH (SA analog), water (negative control)

Sampling times:

0h, 8h, 24h

**7- Study by Chandran et al. (2010) [**[**7**](#_ENREF_7)**]**

Number of Microarrays: 16

Objectives:

To elucidate host processes and components required for the sustained growth and reproduction of the obligate biotrophic fungus *Golovinomyces orontii*, combined with laser microdissection to enrich for genes differentially expressed in response to infection.

Challenges:

*Golovinomyces orontii*

Sampling times:

5 days

**8- Study by Goda et al. (2008) [**[**8**](#_ENREF_8)**]**

Number of Microarrays: 96

Objectives:

AtGenExpress project. The experimental agents included seven basic phytohormones (auxin, cytokinin, gibberellin, brassinosteroid, abscisic acid, jasmonate and ethylene) and their inhibitors. All of the above hormones are implicated in an intricate cross-talk with plant immune signaling pathways, with auxin, jasmonic acid and ethylene being the best known antagonizers of defense to biotrophic pathogens.

Challenges:

Indole-3-acetic acid (IAA) as auxin, trans-zeatin as cytokinin, gibberellin, brassinosteroid, abscisic acid, jasmonate and 1-aminocycropropane-1-carboxylic acid (ACC) as ethylene

Sampling times:

Varied for different hormonal treatments: 0, 30 min, 1h, 3h, 6h, 9h, 24h

**Study by Thilmony et al. (2006) [**[**9**](#_ENREF_9)**]**

Number of Microarrays: 40

Objectives:

Genome-wide gene expression analysis of Arabidopsis plants treated with defined Pst DC3000 and E. coli O157:H7 mutants to gain molecular insights into (i) the transcriptional changes associated with basal defense to live bacteria, which carry multiple MAMPs, (ii) the contribution of flagellin to the regulation of the basal defense transcriptome during infection, and (iii) the global effects of hrp-regulated virulence factors, primarily the TTSS and COR, on the basal defense transcriptome and other host physiological processes.

Challenges:

Pst mutants fliC, *COR^-^* and *hrp^-^* and *E. coli TUV-86-2 fliC*

Sampling times:

0h, 7h, 10h, 24h

**References**

1. Truman W, de Zabala MT, Grant M: **Type III effectors orchestrate a complex interplay between transcriptional networks to modify basal defence responses during pathogenesis and resistance**. *The Plant journal : for cell and molecular biology* 2006, **46**(1):14-33.

2. Zipfel C, Kunze G, Chinchilla D, Caniard A, Jones JD, Boller T, Felix G: **Perception of the bacterial PAMP EF-Tu by the receptor EFR restricts Agrobacterium-mediated transformation**. *Cell* 2006, **125**(4):749-760.

3. Denoux C, Galletti R, Mammarella N, Gopalan S, Werck D, De Lorenzo G, Ferrari S, Ausubel FM, Dewdney J: **Activation of defense response pathways by OGs and Flg22 elicitors in Arabidopsis seedlings**. *Molecular plant* 2008, **1**(3):423-445.

4. Eulgem T, Weigman VJ, Chang HS, McDowell JM, Holub EB, Glazebrook J, Zhu T, Dangl JL: **Gene expression signatures from three genetically separable resistance gene signaling pathways for downy mildew resistance**. *Plant physiology* 2004, **135**(2):1129-1144.

5. Ramonell K, Berrocal-Lobo M, Koh S, Wan J, Edwards H, Stacey G, Somerville S: **Loss-of-function mutations in chitin responsive genes show increased susceptibility to the powdery mildew pathogen Erysiphe cichoracearum**. *Plant physiology* 2005, **138**(2):1027-1036.

6. Wang D, Amornsiripanitch N, Dong X: **A genomic approach to identify regulatory nodes in the transcriptional network of systemic acquired resistance in plants**. *PLoS pathogens* 2006, **2**(11):e123.

7. Chandran D, Inada N, Hather G, Kleindt CK, Wildermuth MC: **Laser microdissection of Arabidopsis cells at the powdery mildew infection site reveals site-specific processes and regulators**. *Proceedings of the National Academy of Sciences of the United States of America* 2010, **107**(1):460-465.

8. Goda H, Sasaki E, Akiyama K, Maruyama-Nakashita A, Nakabayashi K, Li W, Ogawa M, Yamauchi Y, Preston J, Aoki K *et al*: **The AtGenExpress hormone and chemical treatment data set: experimental design, data evaluation, model data analysis and data access**. *The Plant journal : for cell and molecular biology* 2008, **55**(3):526-542.

9. Thilmony R, Underwood W, He SY: **Genome-wide transcriptional analysis of the Arabidopsis thaliana interaction with the plant pathogen Pseudomonas syringae pv. tomato DC3000 and the human pathogen Escherichia coli O157:H7**. *The Plant journal : for cell and molecular biology* 2006, **46**(1):34-53.
