# Supplementary figures and images for: Expression-based network biology identifies immune-related functional modules involved in plant defense
Source: BMC Genomics. 2014 Jun 3;15:421. doi: 10.1186/1471-2164-15-421 (PMC4070563; doi:10.1186/1471-2164-15-421)

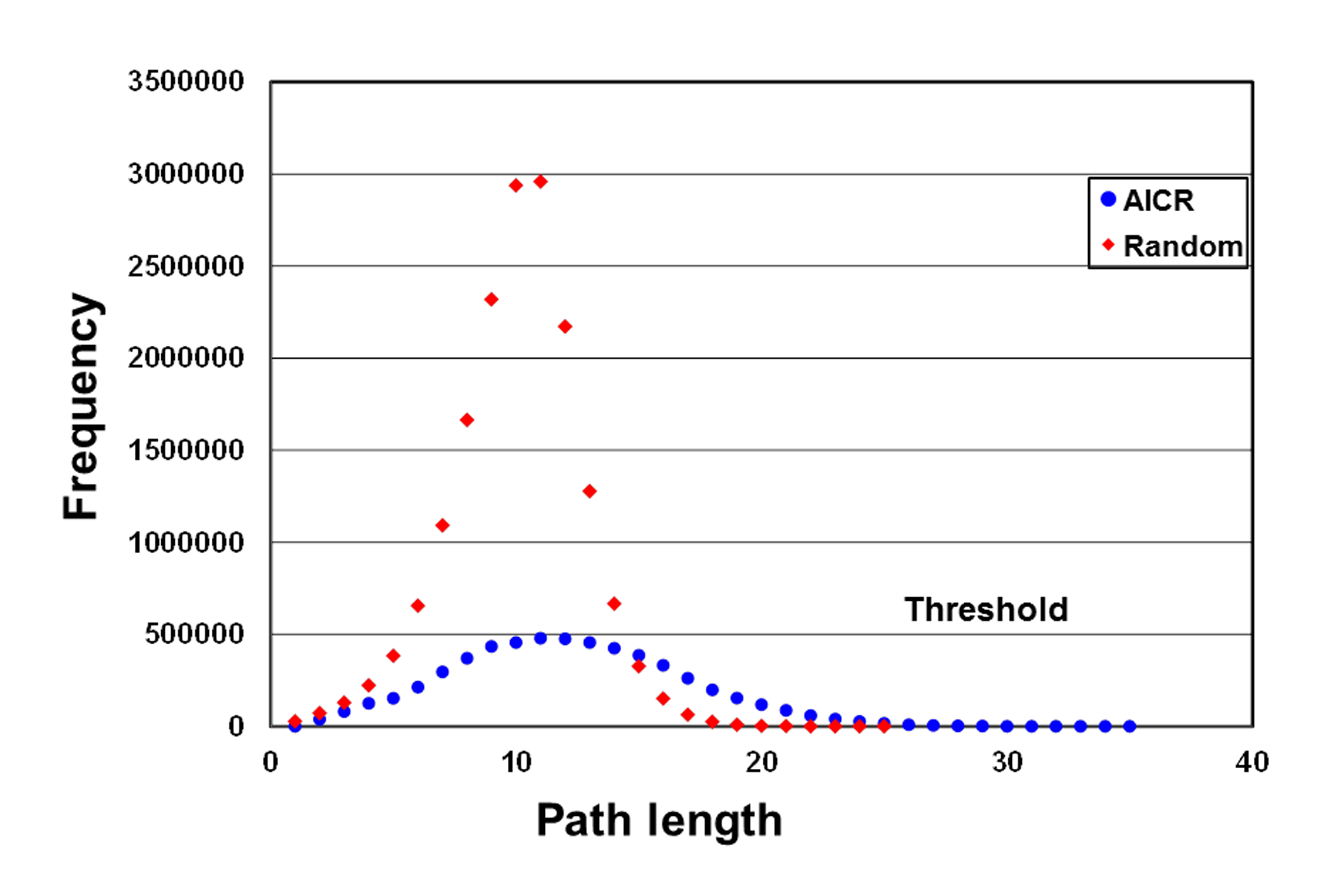

Supplement: Supplementary file 4 — Additional file 4: Figure S1: Distribution of shortest paths in the AICR (blue circles) and random (red diamonds) networks. (TIFF 707 KB) [file 12864_2013_6122_MOESM4_ESM.tiff]

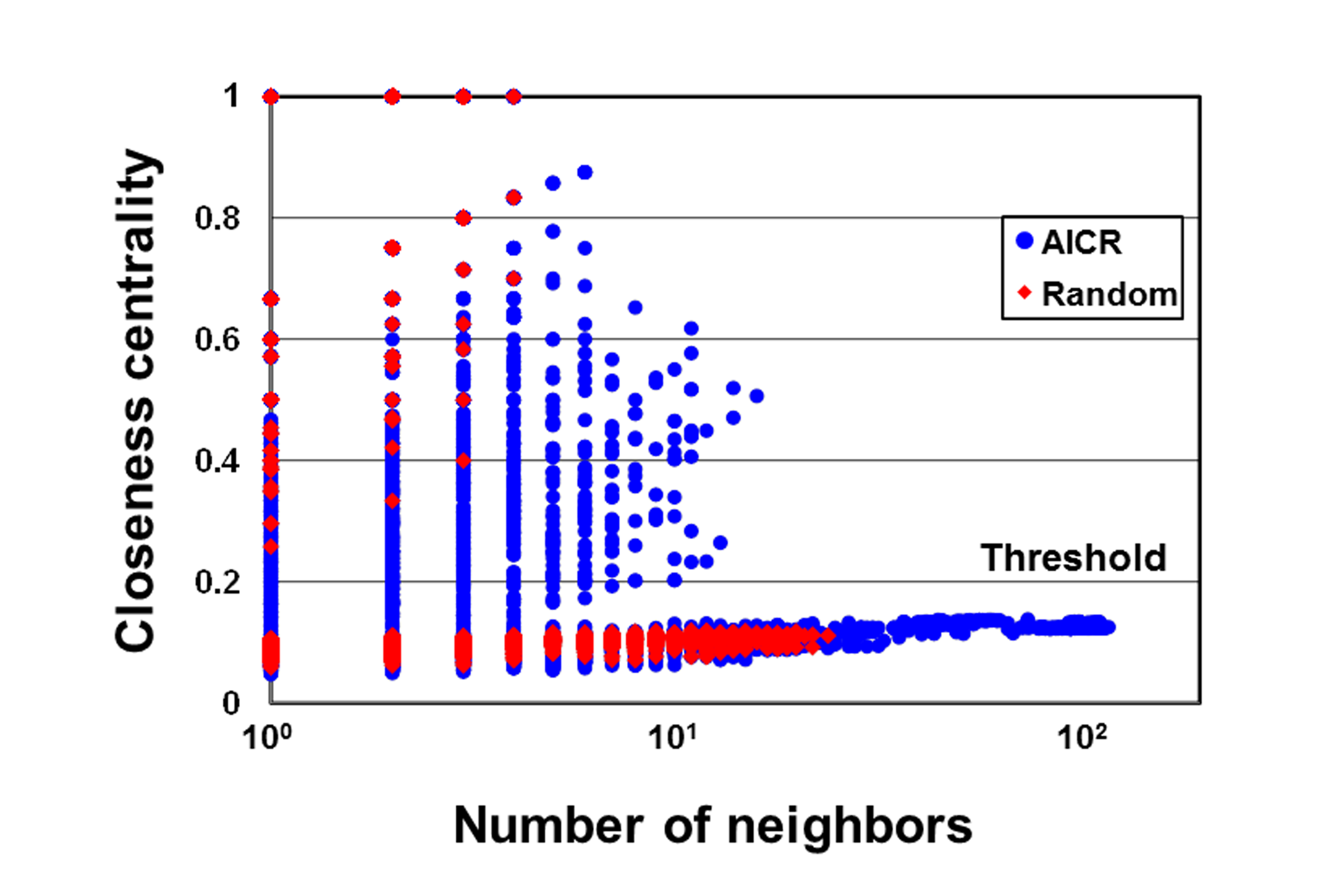

Supplement: Supplementary file 5 — Additional file 5: Figure S2: Closeness centrality property of the AICR network. Distribution of closeness centrality in the AICR (blue circles) and random (red diamonds) networks. (TIFF 1 MB) [file 12864_2013_6122_MOESM5_ESM.tiff]

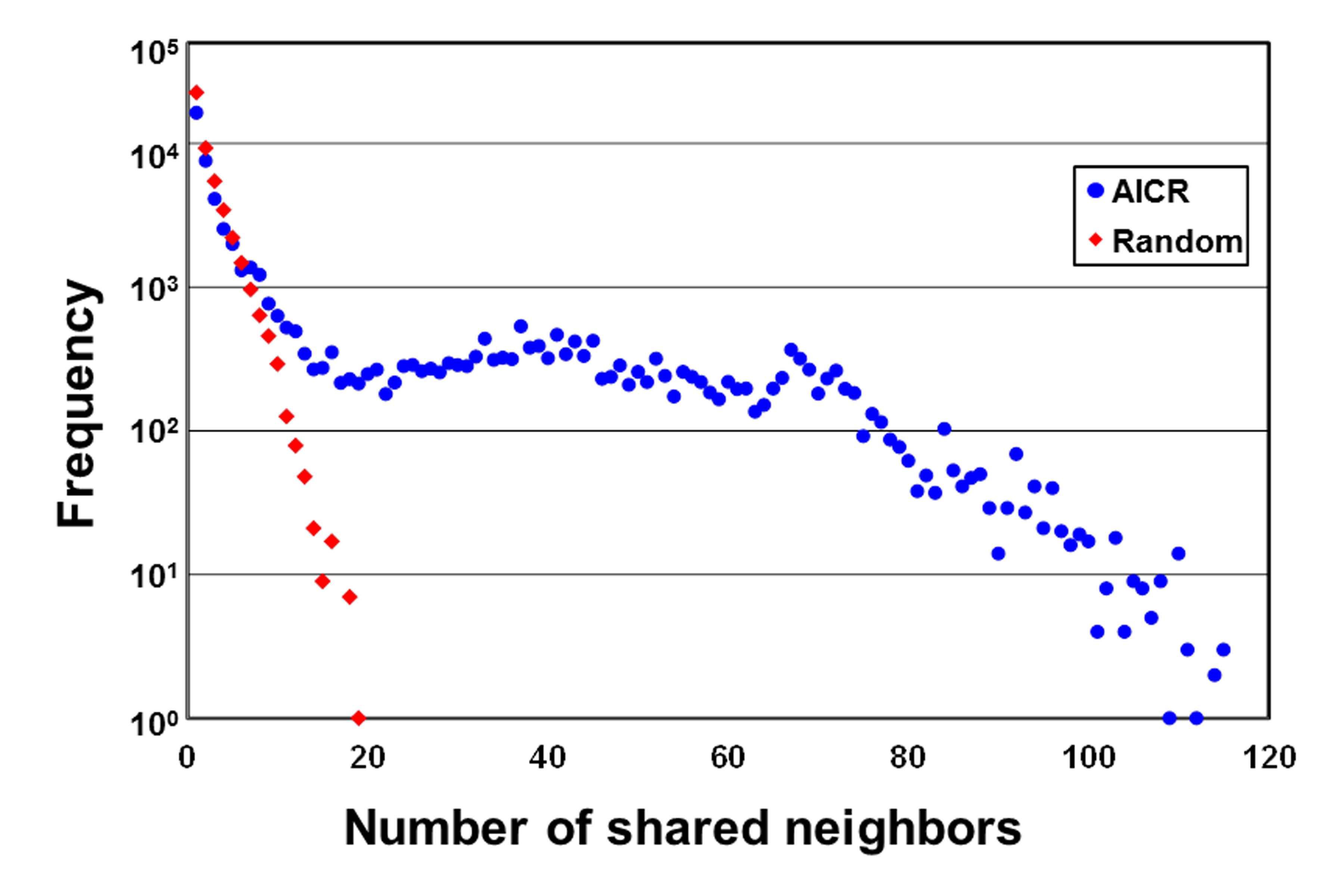

Supplement: Supplementary file 6 — Additional file 6: Figure S3: Evaluation of frequency of number of shared neighbors in the AICR (blue circles) and random (red diamonds) networks. (TIFF 884 KB) [file 12864_2013_6122_MOESM6_ESM.tiff]

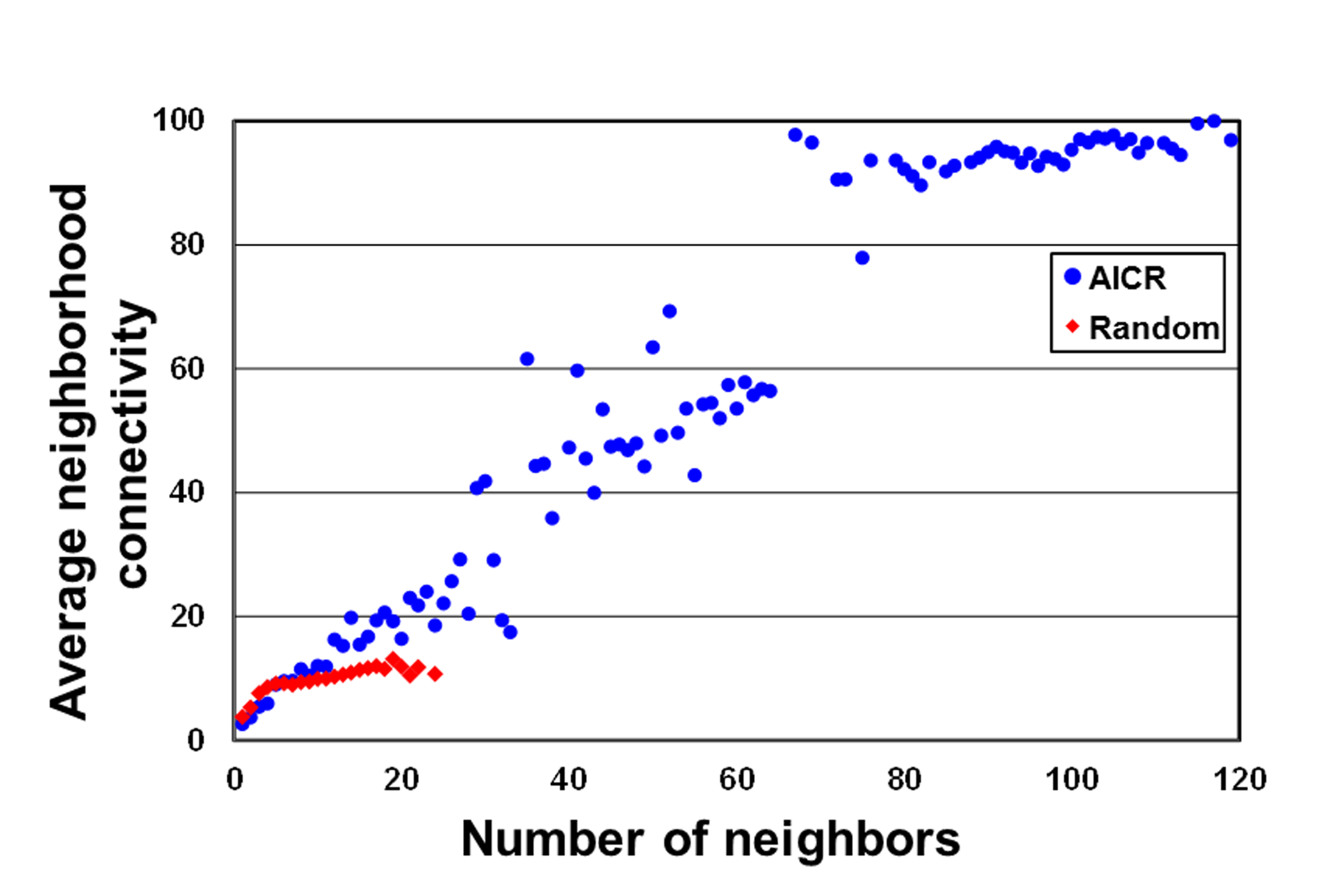

Supplement: Supplementary file 7 — Additional file 7: Figure S4: Determination of neighborhood connectivity frequency in the AICR (blue circles) and random (red diamonds) networks. (TIFF 774 KB) [file 12864_2013_6122_MOESM7_ESM.tiff]

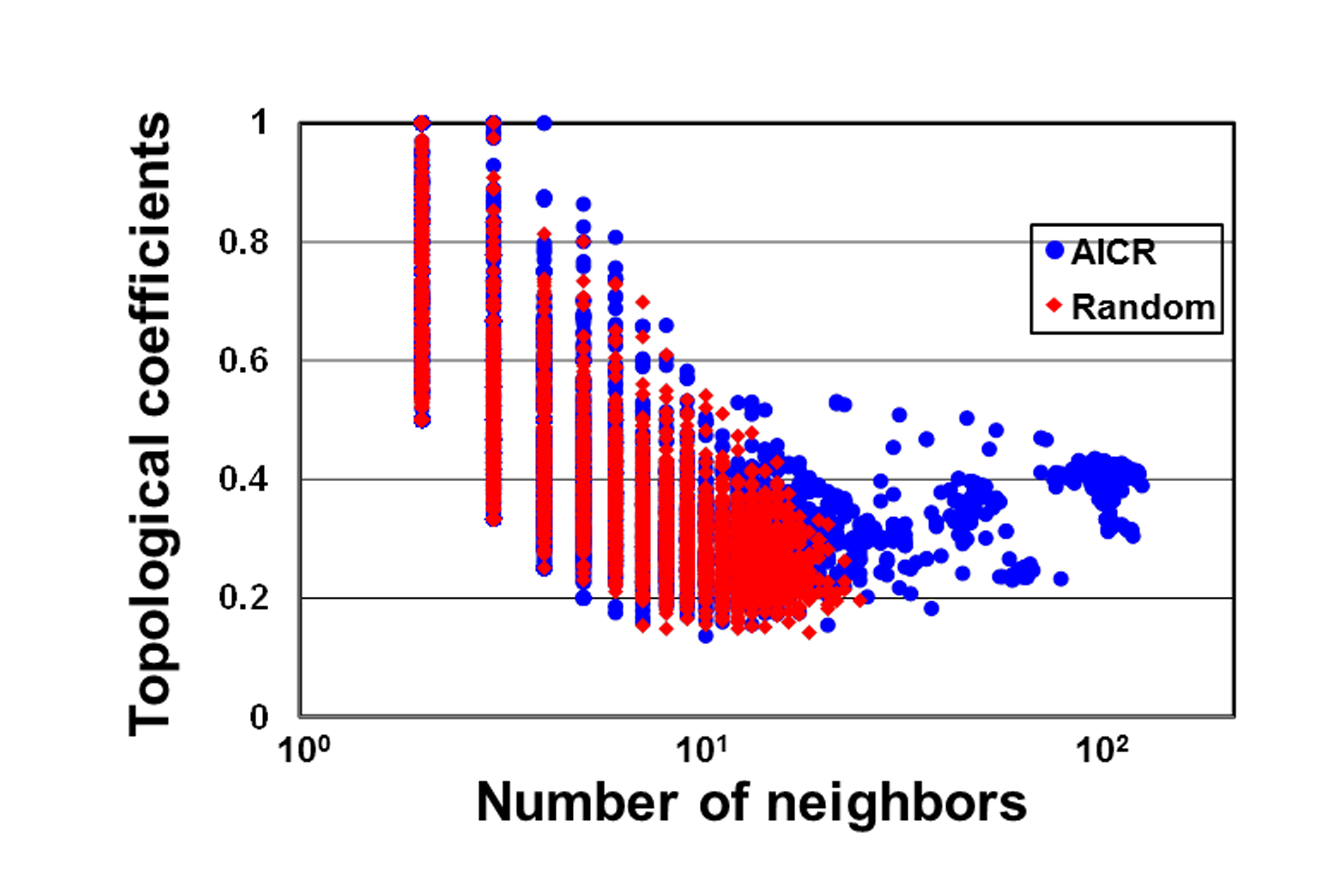

Supplement: Supplementary file 8 — Additional file 8: Figure S5: Distribution of topological coefficients in the AICR (blue circles) and random (red diamonds) networks. (TIFF 2 MB) [file 12864_2013_6122_MOESM8_ESM.tiff]

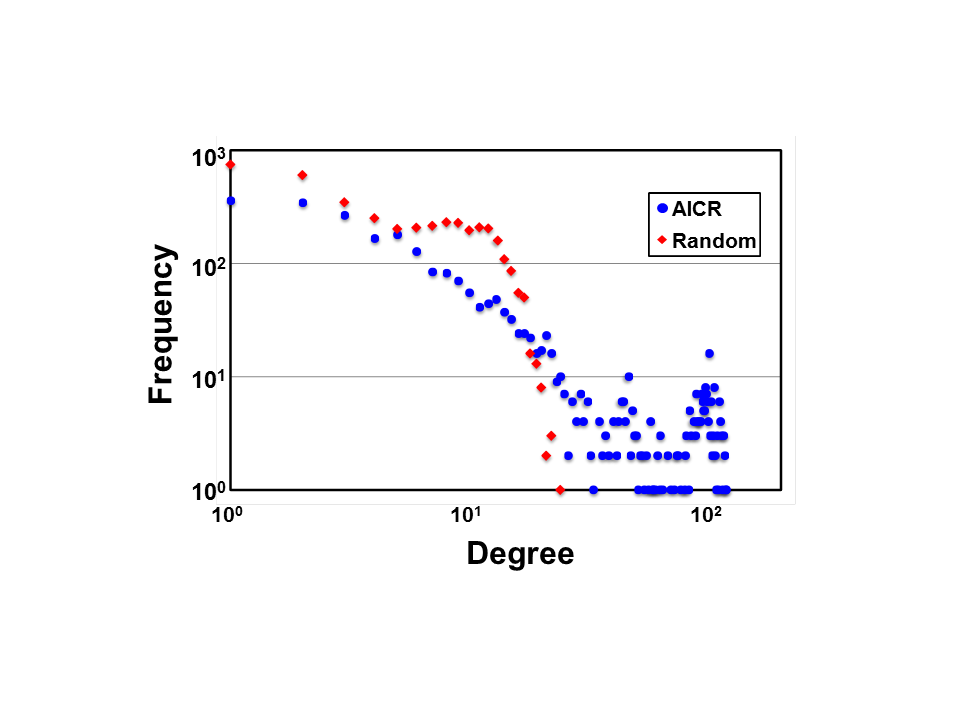

Supplement: Supplementary file 9 — Additional file 9: Figure S6: Degree distributions of main components in the AICR (blue circles) and random (red diamonds) networks. (TIFF 56 KB) [file 12864_2013_6122_MOESM9_ESM.tiff]

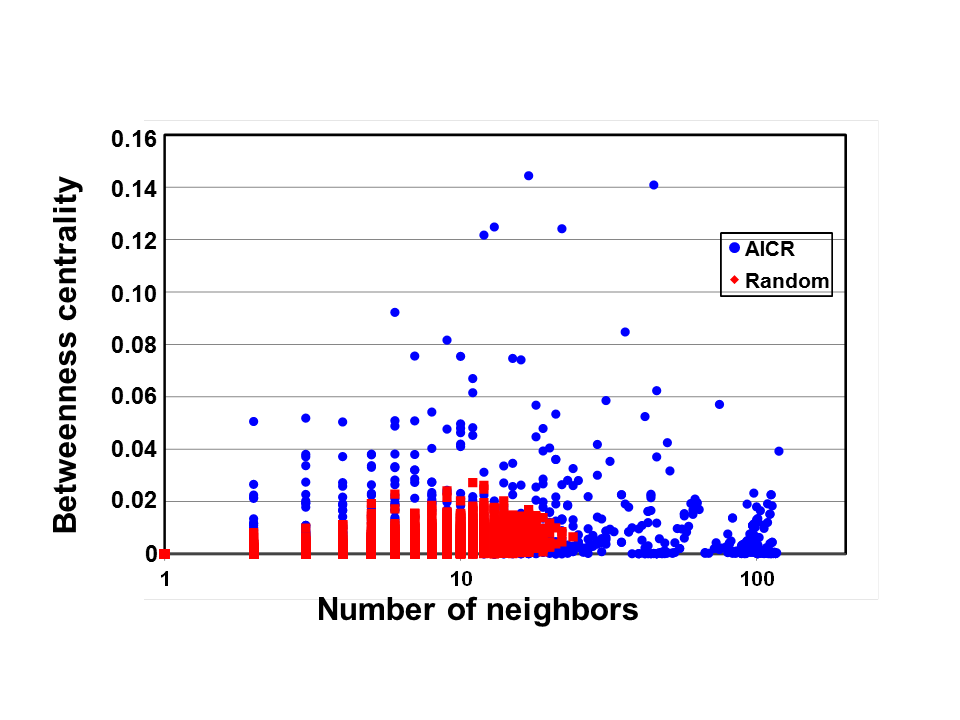

Supplement: Supplementary file 10 — Additional file 10: Figure S7: Betweenness centrality of main components in the AICR (blue circles) and random (red diamonds) networks. (TIFF 57 KB) [file 12864_2013_6122_MOESM10_ESM.tiff]

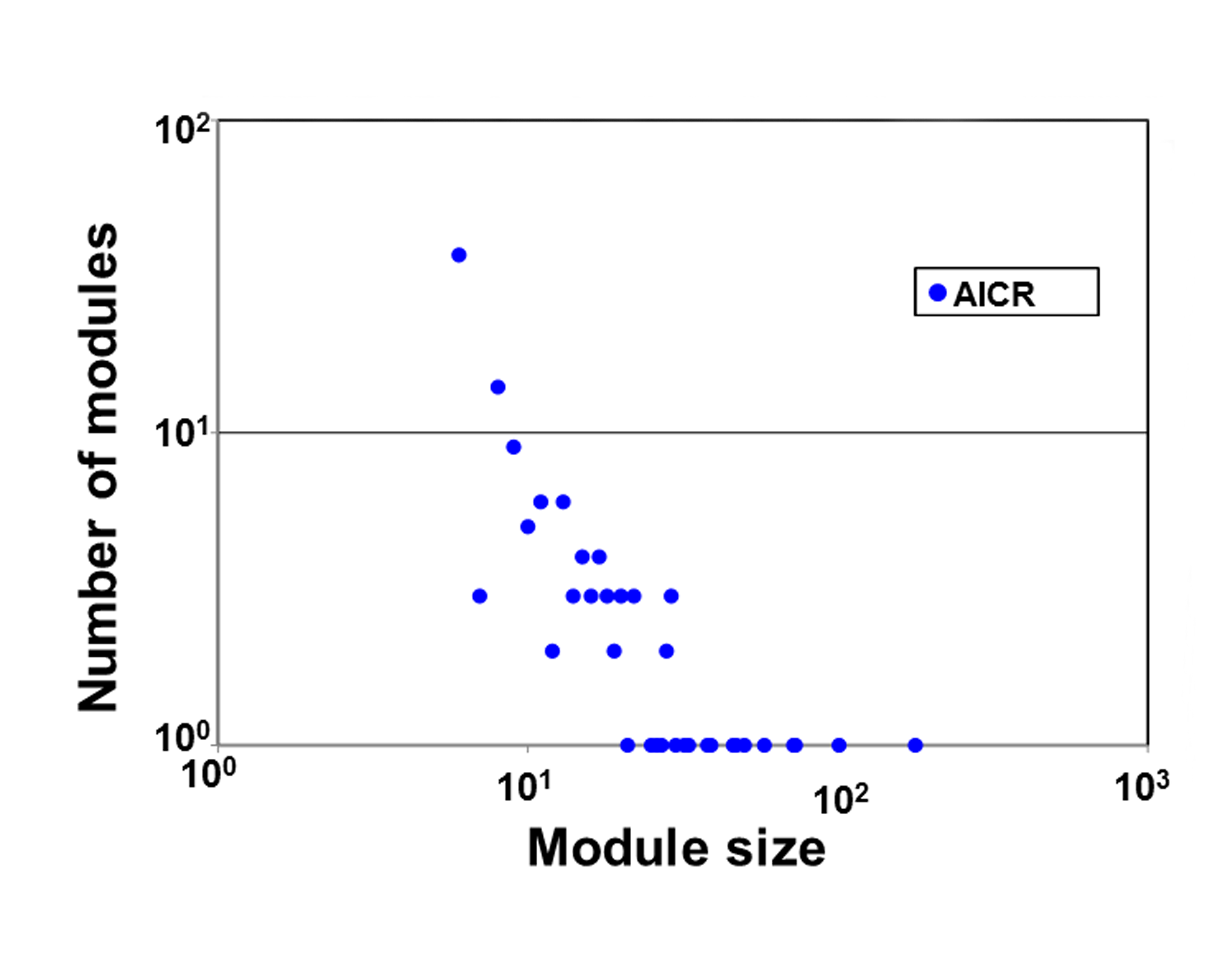

Supplement: Supplementary file 12 — Additional file 12: Figure S8: Distribution of module size in the AICR network. Frequency of module size in the AICR (blue circles) network is shown in log scale. The AICR network exhibits a power law distribution, a network property shared by ‘real-world networks’. (TIFF 462 KB) [file 12864_2013_6122_MOESM12_ESM.tiff]
