# Supplementary material for: Expression-based network biology identifies immune-related functional modules involved in plant defense
Source: BMC Genomics. 2014 Jun 3;15:421. doi: 10.1186/1471-2164-15-421 (PMC4070563; doi:10.1186/1471-2164-15-421)
Supplement: Supplementary file 15 — Additional file 15: Supporting results Module 8 in Arabidopsis immune co-expression regulatory (AICR) network. (DOCX 34 KB) [file 12864_2013_6122_MOESM15_ESM.docx]

**Additional file 13: Supporting results** Module 8 in Arabidopsis immune co-expression regulatory (AICR) network

Module 8 represents a relatively smaller and more compact group of genes, consisting of 46 nodes, 488 edges, clustering coefficient of 0.784 and network density of 0.236. The genes of Module 8 are enriched in two major GO categories: (1) phosphorus metabolic process (2) hormone stimulus (Figure 7, Additional file 9: Table S3 and Additional file 10: Table S4).

The most significantly enriched sub-cluster of Module 8 comprises six genes associated with phosphorus metabolic process. Among these, we found genes enzymes such as an ATP synthase, glycerol-3-phosphate dehydrogenase, isopropylmalate isomerase 1 (IPMI1) and signal peptide cleaving enzyme presequence protease 2 (PREP2). Our data indicate that the balance of metabolic-encoded proteins changes in response to developmental and environmental inputs, in agreement with a previous report on transcriptional reprogramming in Arabidopsis plants treated with immune stimuli, resulting in down-regulation of photosynthesis and transition from growth to defense [[1](#_ENREF_1)].

In addition, we discovered another small cluster of seven genes coding for hormone stimulus. Predominantly, this sub-cluster contains zinc finger family proteins, many of which play critical roles in many cellular functions, including transcriptional regulation, RNA binding, regulation of apoptosis, and protein-protein interactions [[2](#_ENREF_2)]. One of the zinc finger genes identified in Module 8, XERICO, encodes a small protein with an N-terminal transmembrane domain and a RING-H2 zinc finger, which is involved in ABA metabolism and transcriptionally controlled by gibberellic acid signaling components, DELLA proteins [[3](#_ENREF_3), [4](#_ENREF_4)]. We also found a member of the cytochrome P450 family, brassinosteroid-6-oxidase 2 (BR6OX2) also known as CYP85A2 [[5](#_ENREF_5), [6](#_ENREF_6)]. P450 enzymes are haem-thiolate proteins that are widely distributed in bacteria, fungi, plants, and animals. Several members of the cytochrome P450 family were shown to be implicated in plant defense responses, catalyzing the biosynthesis of numerous exogenous and endogenous pro-defense compounds [[5](#_ENREF_5), [6](#_ENREF_6)]. In combination with these published reports, our data indicate a mechanism by which the plant prioritizes pro-defense-related metabolic activities over growth/development [[5-9](#_ENREF_5)].

Consistently with our findings for Module 1, promoters of genes in Module 8 are also enriched in the *APETALA3* binding sites. In addition, we identified presence of the Circadian Clock-Associated 1 (CCA1) binding sequence in the promoters of Module 8 genes. Besides its well-known targets involved in circadian clock control, CCA1 was also recently shown to be implicated in plant defense against oomycete *H. arabidopsidis* [[10](#_ENREF_10)]. CCA1 directly regulates a TIR-NB-LRR resistance gene *RPP4* (*RESISTANCE TO PERONOSPORA PARASITICA 4*) and is postulated to control additional immune-related genes [[10](#_ENREF_10)].

**References:**

1. Pajerowska-Mukhtar KM, Wang W, Tada Y, Oka N, Tucker CL, Fonseca JP, Dong X: **The HSF-like transcription factor TBF1 is a major molecular switch for plant growth-to-defense transition**. *Current biology : CB* 2012, **22**(2):103-112.

2. Ciftci-Yilmaz S, Mittler R: **The zinc finger network of plants**. *Cellular and molecular life sciences : CMLS* 2008, **65**(7-8):1150-1160.

3. Choi H, Hong J, Ha J, Kang J, Kim SY: **ABFs, a family of ABA-responsive element binding factors**. *The Journal of biological chemistry* 2000, **275**(3):1723-1730.

4. Jensen MK, Hagedorn PH, de Torres-Zabala M, Grant MR, Rung JH, Collinge DB, Lyngkjaer MF: **Transcriptional regulation by an NAC (NAM-ATAF1,2-CUC2) transcription factor attenuates ABA signalling for efficient basal defence towards Blumeria graminis f. sp. hordei in Arabidopsis**. *The Plant journal : for cell and molecular biology* 2008, **56**(6):867-880.

5. Bednarek P: **Chemical warfare or modulators of defence responses - the function of secondary metabolites in plant immunity**. *Current opinion in plant biology* 2012, **15**(4):407-414.

6. Hiruma K, Fukunaga S, Bednarek P, Pislewska-Bednarek M, Watanabe S, Narusaka Y, Shirasu K, Takano Y: **Glutathione and tryptophan metabolism are required for Arabidopsis immunity during the hypersensitive response to hemibiotrophs**. *Proceedings of the National Academy of Sciences of the United States of America* 2013, **110**(23):9589-9594.

7. Bednarek P, Pislewska-Bednarek M, Svatos A, Schneider B, Doubsky J, Mansurova M, Humphry M, Consonni C, Panstruga R, Sanchez-Vallet A *et al*: **A glucosinolate metabolism pathway in living plant cells mediates broad-spectrum antifungal defense**. *Science* 2009, **323**(5910):101-106.

8. Clay NK, Adio AM, Denoux C, Jander G, Ausubel FM: **Glucosinolate metabolites required for an Arabidopsis innate immune response**. *Science* 2009, **323**(5910):95-101.

9. Millet YA, Danna CH, Clay NK, Songnuan W, Simon MD, Werck-Reichhart D, Ausubel FM: **Innate immune responses activated in Arabidopsis roots by microbe-associated molecular patterns**. *The Plant cell* 2010, **22**(3):973-990.

10. Wang W, Barnaby JY, Tada Y, Li H, Tor M, Caldelari D, Lee DU, Fu XD, Dong X: **Timing of plant immune responses by a central circadian regulator**. *Nature* 2011, **470**(7332):110-114.
